# Draft genome sequences of the oomycete *Pythium insidiosum* strain CBS 573.85 from a horse with pythiosis and strain CR02 from the environment

**DOI:** 10.1016/j.dib.2017.11.002

**Published:** 2017-11-07

**Authors:** Preecha Patumcharoenpol, Thidarat Rujirawat, Tassanee Lohnoo, Wanta Yingyong, Nongnuch Vanittanakom, Weerayuth Kittichotirat, Theerapong Krajaejun

**Affiliations:** a Systems Biology and Bioinformatics Research Group, Pilot Plant Development and Training Institute, King Mongkut's University of Technology Thonburi, Bangkok, Thailand; b Department of Biomedical Informatics, University of Arkansas for Medical Sciences, Little Rock, AR, USA; c Research Center, Faculty of Medicine, Ramathibodi Hospital, Mahidol University, Bangkok, Thailand; d Department of Microbiology, Faculty of Medicine, Chiang Mai University, Chiang Mai, Thailand; e Department of Pathology, Faculty of Medicine, Ramathibodi Hospital, Mahidol University, Bangkok, Thailand

**Keywords:** *Pythium insidiosum*, Pythiosis, Draft genome sequence

## Abstract

*Pythium insidiosum* is an aquatic oomycete microorganism that causes the fatal infectious disease, pythiosis, in humans and animals. The organism has been successfully isolated from the environment worldwide. Diagnosis and treatment of pythiosis is difficult and challenging. Genome sequences of *P. insidiosum*, isolated from humans, are available and accessible in public databases. To further facilitate biology-, pathogenicity-, and evolution-related genomic and genetic studies of *P. insidiosum*, we report two additional draft genome sequences of the *P. insidiosum* strain CBS 573.85 (35.6 Mb in size; accession number, BCFO00000000.1) isolated from a horse with pythiosis, and strain CR02 (37.7 Mb in size; accession number, BCFR00000000.1) isolated from the environment.

**Specifications Table**

**Table:** 

Subject area	Biology
More specific subject area	Microbiology, Genomics
Type of data	Genome sequence data
How data was acquired	IlluminaHiSeq 2000 and IlluminaHiSeq 2500 Next Generation Sequencing Platforms
Data format	Assembled genome sequences
Experimental factors	Genomic DNA was extracted from the *Pythium insidiosum* strains CBS 573.85 (an animal isolate) and CR02 (an environmental isolate).
Experimental features	Genome of the *P. insidiosum* strains CBS 573.85 and CR02 were sequenced and assembled.
Data source location	*Pythium insidiosum* strain CBS 573.85 was isolated from a horse in Costa Rica, and strain CR02 was isolated from the environment in Thailand.
Data accessibility	The draft genome sequences of *P. insidiosum* have been deposited in the DNA Data Bank of Japan (DDBJ) under the accession numbers: BCFO00000000.1 (strain CBS573.85; https://www.ncbi.nlm.nih.gov/nuccore/BCFO00000000.1) and BCFR00000000.1 (strain CR02; https://www.ncbi.nlm.nih.gov/nuccore/BCFR00000000.1).

**Value of the data**

• Previously, only genome sequence data of *P. insidiosum* isolated from humans is available in the public databases.
• The first draft genome sequences of *P. insidiosum* isolated from a non-human animal with pythiosis and from the environment are now made available.
• The additional genome data will facilitate biology-, pathogenicity-, and evolution-related studies of *P. insidiosum*, through comparative genomic studies of *Pythium* species and related species.

## 1 Data

*Pythium insidiosum* is an aquatic oomycete microorganism that causes the lethal infectious condition, pythiosis, in humans and other animals [1], [2]. The organism has been isolated from the environment in Australia, Thailand, Brazil and the United States [3], [4], [5], [6]. Genome sequences of *P. insidiosum*, isolated from humans, are available and accessible in public databases [7], [8]. We report two additional draft genome sequences of the organism isolated from a horse with pythiosis, as well as from the environment.

## 2 Experimental design, materials and methods

### 2.1 Genome sequencing and assembly

Genomic DNA samples were extracted from *P. insidiosum* strain CBS573.85 (from an infected horse in Costa Rica) and strain CR02 (from an agricultural area in Thailand), using the conventional extraction method described by Lohnoo and co-workers [9]. rDNA sequence analysis was performed to confirm the identity of the organism [10], [11], [12]. The extracted genomic DNA of each of these two strains was subjected to preparation of a paired-end library for genome sequencing, using the IlluminaHiSeq 2500 (strain CBS573.85) or IlluminaHiSeq 2000 (strain CR02) platform (Yourgene Bioscience, Taiwan). Quality trims of the raw reads were executed by CLC Genomics Workbench (www.clcbio.com) to yield read lengths with at least 35 bases. The adaptor sequences were removed by Cutadapt 1.8.1 [13] to obtain a total of 34,651,034 reads with an average read length of 122 bases (4,238,414,330 total bases) for the strain CBS573.85, and a total of 27,436,541 reads with an average read length of 105 bases (2,888,290,738 total bases) for the strain CR02. All reads were assembled by Velvet 1.2.10 [14] to the total sequence length of 35,561,321 bases (number of contigs, 11,223; average contig length, 3169; *N*_50_, 12,261; ‘N’ composition, 1.2%) for the strain CBS573.85, and 37,673,126 bases (number of contigs, 22,560; average contig length, 1670; *N*_50_, 3553; ‘N’ composition, 2.7%) for the strain CR02. CEGMA analysis with 248 highly-conserved eukaryotic genes [15], [16] reported 87% and 77% completeness of draft genomes of the strains CBS573.85 and CR02, respectively. MAKER2 [17] assigned 14,487 (strain CBS573.85) and 15,231 (strain CR02) open reading frames.

### 2.2 Data accessibility

The genome sequence data has been deposited in DDBJ under the accession numbers BCFO00000000.1 (strain CBS573.85) and BCFR00000000.1 (strain CR02).

## References

[1] Krajaejun T., Sathapatayavongs B., Pracharktam R., Nitiyanant P., Leelachaikul P., Wanachiwanawin W., Chaiprasert A., Assanasen P., Saipetch M., Mootsikapun P., Chetchotisakd P., Lekhakula A., Mitarnun W., Kalnauwakul S., Supparatpinyo K., Chaiwarith R., Chiewchanvit S., Tananuvat N., Srisiri S., Suankratay C., Kulwichit W., Wongsaisuwan M., Somkaew S. (2006). Clinical and epidemiological analyses of human pythiosis in Thailand. Clin. Infect. Dis..

[2] Gaastra W., Lipman L.J.A., De Cock A.W.A.M., Exel T.K., Pegge R.B.G., Scheurwater J., Vilela R., Mendoza L. (2010). *Pythium insidiosum*: an overview. Vet. Microbiol..

[3] Miller R.I. (1983). Investigations into the biology of three “phycomycotic” agents pathogenic for horses in Australia. Mycopathologia..

[4] Supabandhu J., Fisher M.C., Mendoza L., Vanittanakom N. (2008). Isolation and identification of the human pathogen *Pythium insidiosum* from environmental samples collected in Thai agricultural areas. Med. Mycol..

[5] Presser J.W., Goss E.M. (2015). Environmental sampling reveals that *Pythium insidiosum* is ubiquitous and genetically diverse in North Central Florida. Med. Mycol..

[6] Zambrano C.G., Fonseca A.O.S., Valente J.S.S., Braga C.Q., Sallis E.S.V., Azevedo M.I., Weiblen C., Santurio J.M., Botton S.A., Pereira D.I.B. (2017). Isolation and characterization of Pythium species from swampy areas in the Rio Grande do Sul, Brazil, and evaluation of pathogenicity in an experimental model. Pesqui. Veter. Bras..

[7] Ascunce M.S., Huguet-Tapia J.C., Braun E.L., Ortiz-Urquiza A., Keyhani N.O., Goss E.M. (2016). Whole genome sequence of the emerging oomycete pathogen *Pythium insidiosum* strain CDC-B5653 isolated from an infected human in the USA. Genome Data.

[8] Rujirawat T., Patumcharoenpol P., Lohnoo T., Yingyong W., Lerksuthirat T., Tangphatsornruang S., Suriyaphol P., Grenville-Briggs L.J., Garg G., Kittichotirat W., Krajaejun T. (2015). Draft genome sequence of the pathogenic oomycete *Pythium insidiosum* strain Pi-S, isolated from a patient with pythiosis. Genome Announc..

[9] Lohnoo T., Jongruja N., Rujirawat T., Yingyon W., Lerksuthirat T., Nampoon U., Kumsang Y., Onpaew P., Chongtrakool P., Keeratijarut A., Brandhorst T.T., Krajaejun T. (2014). Efficiency comparison of three methods for extracting genomic DNA of the pathogenic oomycete *Pythium insidiosum*. J. Med. Assoc. Thail..

[10] Rujirawat T., Sridapan T., Lohnoo T., Yingyong W., Kumsang Y., Sae-Chew P., Tonpitak W., Krajaejun T. (2017). Single nucleotide polymorphism-based multiplex PCR for identification and genotyping of the oomycete *Pythium insidiosum* from humans, animals and the environment. Infect. Genet. Evol..

[11] Badenoch P.R., Coster D.J., Wetherall B.L., Brettig H.T., Rozenbilds M.A., Drenth A., Wagels G. (2001). *Pythium insidiosum* keratitis confirmed by DNA sequence analysis. Br. J. Ophthalmol..

[12] Chaiprasert A., Krajaejun T., Pannanusorn S., Prariyachatigul C., Wanachiwanawin W., Sathapatayavongs B., Juthayothin T., Smittipat N., Vanittanakom N., Chindamporn A. (2009). *Pythium insidiosum* Thai isolates: molecular phylogenetic analysis. Asian Biomed..

[13] Martin M. (2011). Cutadapt removes adapter sequences from high-throughput sequencing reads. EMBnet J..

[14] Zerbino D.R., Birney E. (2008). Velvet: algorithms for de novo short read assembly using de Bruijn graphs. Genome Res..

[15] Parra G., Bradnam K., Ning Z., Keane T., Korf I. (2008). Assessing the gene space in draft genomes. Nucleic Acids Res..

[16] Parra G., Bradnam K., Korf I. (2007). CEGMA: a pipeline to accurately annotate core genes in eukaryotic genomes. Bioinformatics.

[17] Holt C., Yandell M. (2011). MAKER2: an annotation pipeline and genome-database management tool for second-generation genome projects. BMC Bioinform..

